# Expression and Distribution Pattern of Pnn in Ischemic Cerebral Cortex and Cultured Neural Cells Exposed to Oxygen-Glucose Deprivation

**DOI:** 10.3390/brainsci10100708

**Published:** 2020-10-05

**Authors:** Shu-Yuan Hsu, Sujira Mukda, Steve Leu

**Affiliations:** 1 Department of Anatomy, Graduate Institute of Biomedical Sciences, College of Medicine, Chang Gung University, Taoyuan 33302, Taiwan; hsusy@mail.cgu.edu.tw; 2 Institute for Translational Research in Biomedicine, Kaohsiung Chang Gung Memorial Hospital, Kaohsiung 83301, Taiwan; 3 Research Center for Neuroscience, Institute of Molecular Biosciences, Mahidol University, Salaya, Nakhon Pathom 73170, Thailand; sujira.muk@mahidol.edu; 4 Department of Biotechnology, College of Life Science, Kaohsiung Medical University, Kaohsiung 807808, Taiwan

**Keywords:** Pnn, Pinin, oxygen-glucose deprivation and reoxygenation, ischemic stroke

## Abstract

Pinin (Pnn), a multifunctional protein, participates in embryonic development as well as in cellular apoptosis, proliferation, and migration through regulating mRNA alternative splicing and gene transcription. Previous studies have shown that Pnn plays important roles in neural system development and the expression level of Pnn in astrocytes is altered by ischemic stress and associated with cellular apoptosis. In the present study, we further utilized primary cultured rat neurons and astrocytes with oxygen-glucose deprivation (OGD) and a mouse model with middle cerebral artery occlusion (MCAO)-induced ischemic stroke to examine the effect of ischemic stress on Pnn expression and distribution in different types of neural cells. Under normoxia, Pnn is mainly localized in the nuclear speckle of primary cultured neurons. The expression level of Pnn was increased after the OGD treatment and then decreased in the reoxygenation period. Moreover, the cytoplasmic expression of Pnn was observed in neurons with OGD and reoxygenation (OGD/R). Unlike that in neurons, the Pnn expression in astrocytes was decreased after OGD treatment and then gradually increased during the reoxygenation period. Of interest, the nuclear–cytoplasmic translocation of Pnn was not observed in astrocytes with OGD/R. In the MCAO mouse model, the neuronal expression of Pnn in the peri-ischemic region was reduced by three days post induction of ischemic stroke. However, the Pnn expression in astrocytes was not altered. Moreover, the nuclear speckle distribution of Pnn in neurons was also diminished following ischemic stroke. In conclusion, the Pnn expression and distribution after OGD and during reoxygenation showed distinct manners in neurons and astrocytes, implying that Pnn may play different roles in different types of neural cells in the stress response to ischemic injury.

## 1. Introduction

Pinin (Pnn), a multi-functional serine and arginine-rich (SR) related protein, has been demonstrated to play roles in regulating cellular apoptosis, migration, and proliferation in cultured cells. [1,2,3,4,5]. Recent studies have also indicated that Pnn participates in tumorigenesis and embryonic development [6,7,8]. Previous studies have shown that loss of Pnn alters the expression levels of splicing factor SRSF-1 (ASF/SF2) and results in cellular apoptosis through modulating mRNA alternative splicing of apoptosis-associated genes [1]. In addition to splicing regulators, Pnn is also found to interact with RNPS1 and histone deacetylase complex subunit (SAP18), subunits of apoptosis and splicing-associated protein (ASAP) complex and participates in nonsense-mediated decay of mRNA [9]. The mouse model with Pnn deficiency also indicated that loss of Pnn results in early embryonic lethality [1]. Interestingly, Pnn deficiency only renders cellular apoptosis in proliferative carcinoma cells but not in normal HUVECs [1], indicating that Pnn may play different roles in different kinds of cells.

Thrombotic or embolic cerebral infarction that are a result of atherosclerotic obstruction or clot-induced embolisms in cervical and cerebral arteries are major types of ischemic stroke, which accounts for approximately 80% of all strokes [10,11]. A few minutes after a focal ischemic stroke occurring, the core of brain tissue without blood flow supply is fatally injured and subsequently undergoes necrotic cell death. Surrounding the necrotic core, there is a zone with less severe ischemia and remains metabolically active [12,13]. This border region, the peri-infarct area, is an opportunity for survival after appropriate post-ischemic stroke therapy [12,13]. Recent research has revealed that neurons in the peri-infarct area may undergo apoptosis after several days and are potentially recoverable for some time after the onset of stroke [14,15]. Hence, the regulation of expression or activation of apoptotic/anti-apoptotic proteins is one possible strategy to ameliorate neuronal injury after ischemic stroke. A recent study indicated that Pnn plays a protective role in astrocytes against ischemic stroke in rats [16], while the expression level of Pnn in astrocytes is associated with mitochondrial function and expression of apoptosis-associated proteins [16]. However, the expression and regulation of Pnn in other type of neural cells, particular in neurons, under ischemic stress remains unknown.

In the present study, we applied the ischemic stroke mouse models to determine the effects of ischemic injury on the expression and distribution patterns of Pnn in cerebrum. To further examine the expression and distribution of Pnn in different type of neural cells, fetal neurons and astrocytes were isolated and cultured in the oxygen-glucose deprivation and reoxygenation (OGD/R) condition to simulate the ischemia-reperfusion in vitro. Histological and biochemical examinations were used to examine the distribution and expression of Pnn in neural cells with ischemic insults.

## 2. Materials and Methods

### 2.1. Ethics

All animal experimental procedures were approved by the Institute of Animal Care and Use Committee at Kaohsiung Chang Gung Memorial Hospital (no. 2015100501) and performed in accordance with the Guide for the Care and Use of Laboratory Animals (NIH publication no. 85-23, National Academy Press, Washington, DC, USA, revised 1996).

### 2.2. Animals and Middle Cerebral Artery Occlusion Operation

Adult C57BL/6 mice, weighing 25–30 g, were anesthetized with 2.0% inhalational isoflurane, supine on a warming pad (37 °C). After exposure of the left common carotid artery (LCCA) through a transverse neck incision, the vessel was permanently ligated. On the other hand, after exposure of the right common carotid artery (RCCA) through a transverse neck incision, a small incision was made on the LCCA through which a nylon filament (0.028 mm in diameter) was carefully advanced into the distal right internal carotid artery for occlusion of the right middle cerebral artery (RMCA) to cause brain infarction of its supplied area. The nylon filament was removed 120 min after occlusion, followed by closure of the muscle and skin in layers.

### 2.3. Culturing of Prenatal Primary Neurons and Astrocytes

The isolation and culturing of primary cortical neurons was performed as previously described [17]. Briefly, primary cortical neurons were prepared from 17-day old Sprague-Dawley rat fetus. Cells were plated in 100-mm diameter polyethyleneimine-coated plastic dishes with minimum essential medium with Earle’s salts supplemented 10% with heat-inactivated fetal bovine serum (FBS) and containing 1 mM L-glutamine, 1 mM pyruvate, 20 mM KCl and 26 mM sodium bicarbonate (pH 7.2). Following cell attachment (3–4 h after plating), the medium was cultured in neurobasal medium containing B-27 supplements (Life Technologies, Inc.), 1 mM HEPES, 2 mM L-glutamine, and 0.001% gentamycin sulfate for 7–9 days. The isolation and culturing of primary astrocytes was performed as previously described [16]. Briefly, the cerebral cortex was aseptically dissected from 17-day old Sprague-Dawley rat fetus and placed in MEM containing 2 mg/mL trypsin and incubated at 37 °C for 30 min. The cortical tissues were mechanically triturated with a pipette until they dissociated into single cells. FBS was then added to stop the activity of trypsin. Cells were then seeded into polyethylenimine-coated culture flask and incubated at 37 °C for 3–4 h until the cells attached. Cells were cultured in high glucose DMEM containing N-2 supplement, 10% heat-inactivated FBS and 1% penicillin/streptomycin.

### 2.4. Western Blot

Protein extracts from cerebral tissues or cells (10–30 μg) were loaded and separated by SDS-PAGE using 8%–12% acrylamide gradients. Following electrophoresis, the separated proteins were transferred electrophoretically to a polyvinylidene difluoride (PVDF) membrane (Amersham Biosciences). Membranes were incubated with blocking buffer (5% nonfat dry milk in T-TBS containing 0.05% Tween 20) to block nonspecific proteins. The membranes were then incubated with primary antibodies against Pnn (P3A, 1:2000, kind gift from Dr. Pin Ouyang), Bax (1:1000, Abcam), annexin V (Abcam, 1:1000), cleaved-caspase 3 (Abcam, 1: 3000), AIF (1:1000 cell signaling), lamin B (Abclone, 1:1000), β-tubulin (Abcam, 1:1000), and SAP18 (1:500, Santa Cruz) for 1 h at room temperature. Signals were detected with HRP-conjugated goat anti-mouse or goat ant-rabbit with ECL (Perkin Elmer, MA, USA).

### 2.5. Histopathological and Immunofluorescent Staining

For immunofluorescent staining, isolated cerebral tissues were mounted in OCT and used for preparing cryosections. Cryosections (10 μm) were fixed and permeated with acetone or 4% paraformaldehyde with 0.5% Triton X-100, and then incubated with antibodies against Pnn (P3A, 1:1000, kind gift from Dr. Pin Ouyang), SAP18 (1:100, Santa Cruz), a neuron nuclear antigen (NeuN) (1:500, Abcam), and GFAP (1:1000, Cell Signaling Technology) at 4 °C overnight. Sections were then incubated with Alex488 or Alex594-conjugated goat anti- mouse or rabbit invitrogen (IgG) for 1 h at room temperature. After counterstaining with DAPI, sections were examined with a fluorescent microscope.

### 2.6. Statistical Analysis

Data were expressed as mean values with standard deviation (mean ± SD). One-way ANOVA was used to evaluate the significance of differences among the groups, followed by Bonferroni multiple comparison post hoc test. Statistical analysis was performed using Prism 7 statistical software (GraphPad Software, La Jolla, CA, USA). A probability value <0.05 was considered statistically significant.

## 3. Results

### 3.1. Oxygen-Glucose Deprivation and Reoxygenation Leads to Distinct Pattern of Pnn Expression in Neurons and Astrocytes

**Figure 1:**
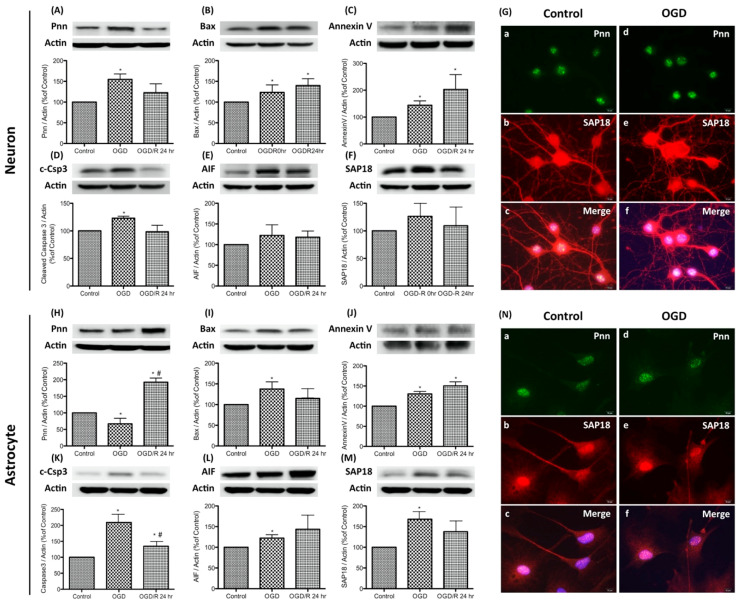
Expression and distribution of Pinin (Pnn) in primary cultured neurons and astrocytes with oxygen-glucose deprivation and reoxygenation. Western blotting was used to examine the protein expression of Pnn, histone deacetylase complex subunit (SAP18), and apoptosis-associated proteins (Bax, cleaved caspase-3, annexin V, caspase-independent apoptosis-inducing factor (AIF)) in neurons (**A**–**F**) and astrocytes (**H**–**M**) with oxygen-glucose deprivation and reoxygenation. Immunofluorescent stainings were used to examine the distribution of Pnn and SAP18 in neurons (**G**) and astrocytes (**N**) with oxygen-glucose deprivation. OGD, oxygen-glucose deprivation; OGD/R, oxygen-glucose deprivation and reoxygenation; AIF; caspase-independent apoptosis-inducing factor. * indicates *p* < 0.05 in comparison with control group; # indicated *p* < 0.05 in comparison with OGD group; *n* = 6 for each group. (**G** and **N**) scale bars in left lower corner of indicate 10 μm.

To simulate the hypoxic-ischemic condition of severe stroke in the in vitro study, oxygen-glucose deprivation (OGD) and reoxygenation was applied to primary neurons and astrocytes. OGD was performed by incubating cells with glucose-free medium in a hypoxic chamber with 5% CO_2_, 1% O_2_, and 94% N_2_ for 24 h. The normoxia control cells were incubated at 37 °C in a humidified atmosphere of 95% air with 5% CO_2_. For reoxygenation, OGD-treated cells were incubated with fresh medium and returned to a 37 °C incubator. Cells were collected immediately after OGD exposure (the OGD group) or with additional reoxygenation for 24 h (the OGD/R group). To determine whether OGD or OGD/R regulates Pnn expression and induces stress response in neurons and astrocytes, expression levels of Pnn and apoptosis-associated proteins, including Bax, cleaved caspase-3 (c-Csp3), annexin V, and caspase-independent apoptosis-inducing factor (AIF) were examined with Western blotting (Figure 1). Results show that the expression of Pnn increased immediately after OGD and reverted post reoxygenation (Figure 1A); however, the expression of Pnn in astrocytes showed a contrary manner (Figure 1H). Furthermore, after OGD treatment, the expression levels of Bax, annexin V, and c-Csp3 were all increased in both neurons and astrocytes (Figure 1B–D,I–K), while only the level of annexin V was further upregulated by reoxygenation (Figure 1C,J). In addition, the expression levels of AIF were also upregulated in OGD-treated neurons and astrocytes (Figure 1E,L).

In addition to Pnn, Western blots and immunofluorescent staining were performed to examine the expression and distribution of SAP18, a Pnn-interacted histone deacetylase complex subunit, in neural cells. Results show that the expression level of SAP18 was upregulated in neurons and astrocytes with OGD treatment (Figure 1F,M), while the distribution patterns of SAP18 was only altered in OGD-treated neurons (Figure 1G), not in OGD-treated astrocytes (Figure 1N). After OGD-treatment, the nuclear localized of SAP18 was significantly reduced in neurons, while more intense nuclear expression of SAP18 was observed in OGD-treated astrocytes. Of interest, although no significant co-localization of Pnn and SAP18 was observed in both neurons and astrocytes under normoxia, the co-localization of Pnn and SAP18 was found in astrocytes with OGD treatment (Figure 1N).

**Figure 2:**
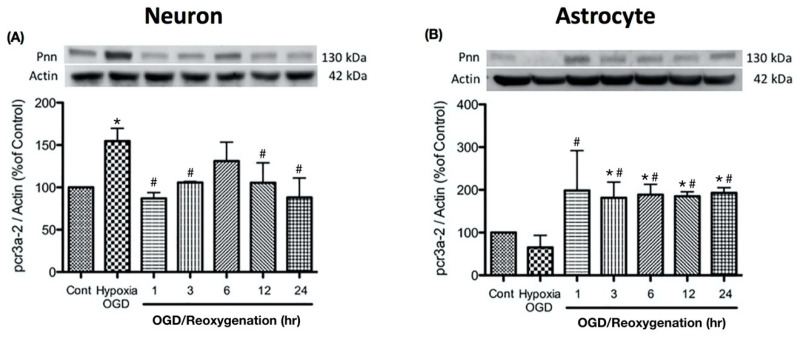
Oxygen-glucose deprivation treatment regulated the expression levels of Pnn in primary cultured cortical neurons (**A**) and astrocytes (**B**). Western blotting was used to examine the expression levels of Pnn after oxygen-glucose deprivation (OGD) treatment and at different time points during reoxygenation. OGD, oxygen-glucose deprivation; * indicates *p* < 0.05 in comparison with control group; # indicated *p* < 0.05 in comparison with OGD group; *n* = 6 for each group.

To examine the alternation of Pnn expression during oxygen-glucose deprivation and reoxygenation in more detail, the expression levels of Pnn at different time points of reoxygenation were examined with Western blot analysis. In primary cortical neurons, the increased level of Pnn was observed after exposure to OGD, while the reoxygenation treatment reduced the OGD-induced upregulation of Pnn (Figure 2A). In astrocytes, OGD treatment significantly decreased the expression of Pnn, while the expression level of Pnn was reverted during the reoxygenation period.

### 3.2. Oxygen-Glucose Deprivation and Reoxygenation Induces the Nuclear–Cytoplasmic Translocation of Pnn in Neurons

**Figure 3:**
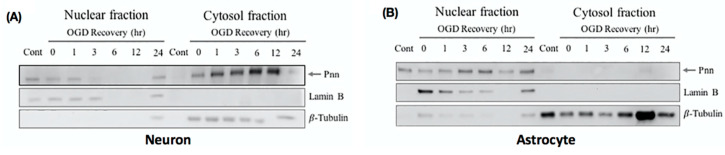
Oxygen-glucose deprivation and reoxygenation induces cytoplasmic translocation of Pnn in neurons. To examine the subcellular localization of Pnn during reoxygenation, cellular fractionation was applied to separate nuclear and cytoplasmic proteins of neurons (**A**) and astrocytes (**B**) at different time points during reoxygenation. Western blotting was performed to examine protein expression levels of Pnn, lamin B (a nuclear envelop protein, nuclear fraction maker), and β-tubulin (a cytoskeleton protein, cytoplasmic fraction marker).

In addition to the expression level, the intracellular translocation also determines the physiological function of proteins. To gain more information regarding the subcellular localization of Pnn after OGD and reoxygenation treatment, cytoplasmic and nuclear fraction of neurons (Figure 3A) and astrocytes (Figure 3B) were used for Western blotting to detect intracellular distribution of Pnn. In neurons with normoxia, Pnn was mainly localized in the nucleus. After exposure to OGD, the cytoplasmic expression level of Pnn was increased with a time-dependent manner during the re-oxygenation period (Figure 3A); however, no translocation of Pnn was observed in astrocytes with OGD and reoxygenation (Figure 3B).

### 3.3. The Cell Type-Specific Distribution of Pnn in the Mouse Cerebrum with Ischemic Stroke

**Figure 4:**
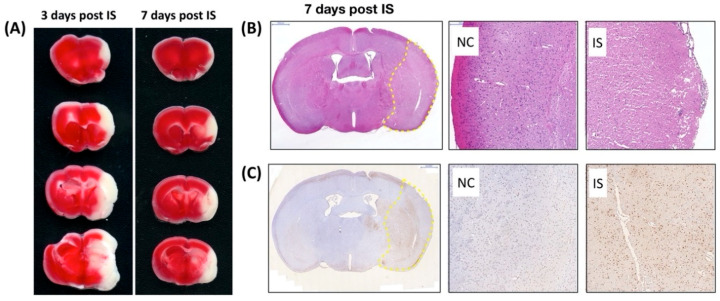
Characterization of cerebral ischemia stroke in mouse. 2,3,5-triphenyltetrazolium chloride (TTC) staining, hematoxylin and eosin (H&E) staining, and terminal deoxynucleotidyl transferase (TdT) dUTP nick-end labeling (TUNEL) assay were used to identify the brain infarcted region in mice with middle cerebral artery occlusion (MCAO). (**A**) The white color shown in the TTC staining indicates the loss of dehydrogenase activity in the infarcted region. (**B**) Seven days post ischemic stroke, to identify the cerebral infarction in high power field, the histopathological examination was performed on cerebral paraffin sections with H&E staining. The infarcted region is indicated by the yellow dashed line surrounding. (**C**) Seven days post ischemic stroke, to validate the apoptosis or necrosis in the infarcted or peri-ischemic region, TUNEL assay was performed on cerebral paraffin section. The dramatically increased apoptotic nuclei (with DNA fragmentation, brown color) were observed in the cerebral striatum and cortex with ischemic stroke. IS, ischemic stroke.

**Figure 5:**
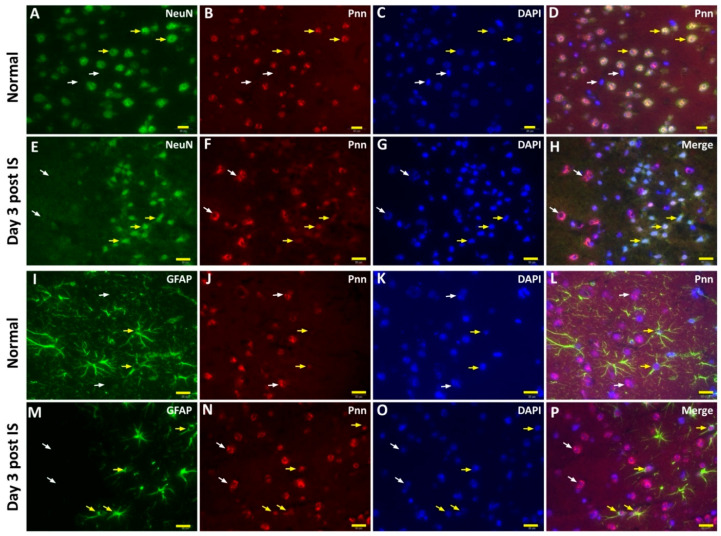
The cell type-specific distribution of Pnn in the mouse cerebrum after induction of ischemic stroke. Immunofluorescent stainings were performed to detect the expression and distribution of Pnn, a neuron nuclear antigen (NeuN), and glial fibrillary acidic protein (GFAP, a marker of astrocyte and ependymal cells) in cerebrum 3 days post ischemic stroke. (**A**–**D**) Distribution of Pnn in neurons of normal control cerebral cortex. (**E**–**H**) Distribution of Pnn in neurons of cerebral cortex with ischemic stroke. (**I**–**L**) Distribution of Pnn in astrocytes of normal control cerebral cortex. (**M**–**P**) Distribution of Pnn in astrocytes of cerebral cortex with ischemic stroke. Yellow arrows in (**A**) to (**H**) indicate the expression of Pnn in NeuN+ neurons. White arrows in (**A**) to (**H**) indicate the expression of Pnn in non-neuron cells. Yellow arrows in (**I**) to (**P**) indicate the expression of Pnn in GFAP+ astrocytes. White arrows in (**I**) to (**P**) indicate the expression of Pnn in non-astrocyte cells. Scale bars in left lower corner of indicate 20 μm.

To directly examine the effect of ischemic stress on regulating the expression and distribution of Pnn in neurons and astrocytes in vivo, the middle cerebral artery occlusion (MCAO) model was applied to induce ischemic stroke in mice. To identify the cerebral infarcted region induced by MCAO, by three and seven days after induction of cerebral ischemic stroke whole brains were isolated for either 2,3,5-triphenyltetrazolium chloride (TTC) staining (Figure 4A), hematoxylin and eosin (H&E) staining (Figure 4B), or terminal deoxynucleotidyl transferase (TdT) dUTP nick-end labeling (TUNEL) assay (Figure 4C). Following histopathological examination, immunofluorescent staining with antibodies against Pnn, a neuron nuclear antigen (NeuN), and glial fibrillary acidic protein (GFAP, a marker of astrocyte and ependymal cells) were performed on cryosections of cerebrum three days post ischemic stroke (Figure 5). In normal condition, Pnn was expressed in both neurons (Figure 5A–D) and astrocytes (Figure 5I–L) in the cerebral cortex. Three days post ischemic stroke, the expression of Pnn in the NeuN positively stained neurons was reduced in the peri-infarcted area (Figure 5E–H). Moreover, the intra-nuclear localization of Pnn was altered, from nuclear speckle to a pan-nucleus pattern (Figure 5E–H). Unlike that in neurons, by three days post ischemic stroke, the Pnn expression and distribution pattern within astrocytes in the peri-infarcted area was not significantly altered (Figure 5M–P). However, along with the loss of Pnn in neurons in the peri-infarcted area, non-neuron and non-astrocyte cells with intense Pnn expression and larger nuclei were observed in the infarcted area (Figure 5E–H,M–P).

## 4. Discussion

In the present study, we have applied the mouse model with ischemic stroke and primary cultured rat neurons and astrocytes with OGD and reoxygenation to examine the effect of ischemic stress on regulating the expression and distribution of Pnn in neural cells. Both in vitro and in vivo experimental results show that the alteration of Pnn expression post OGD and during reoxygenation in neurons is distinct from that observed in astrocytes. OGD stress increased the expression level of Pnn in neurons but reduced the expression in astrocytes, while reoxygenation reduced the expression of Pnn in neurons but upregulated Pnn expression in astrocytes ( Figure 1; Figure 2). Of importance, the OGD/R-induced cytoplasmic translocation of Pnn was only observed in neurons, but not in astrocytes (Figure 3). Similar to that observed in the in vitro cultured cell study, reduced expression of Pnn was observed in neurons in the peri-infarcted region, indicating reperfusion may reduce the expression of Pnn in neurons. However, the expression of Pnn in astrocytes in the peri-infarcted region was not affected by ischemic stress (Figure 5).

Previous studies have shown that Pnn plays essential roles during embryogenesis [1,18], while the participation of Pnn in tumorigenesis and metastasis in certain types of cancers was also reported [6,19,20,21,22]. Although several studies have attempted to reveal the role of Pnn in primary astrocytes and corneal cells [16,23,24], the role and regulation of Pnn in non-dividing cells, particularly in neurons, remains unknown. In this study, we firstly characterized the expression and distribution of Pnn in neurons with the stress response to ischemic insults. A recent study using primary astrocytes indicated that the expression of Pnn is associated with mitochondrial activity and plays a protective role during ischemic stress [16]. In this study, we further demonstrated that the regulation of Pnn expression post ischemic stress in neurons is distinct from that observed in astrocytes.

Pnn is reported to have the ability to modulate pre-mRNA alternative splicing of anti-apoptotic genes [1] and to enhance tumor cell survival under glucose deprivation [7]. It is reasonable that the upregulated expression of Pnn in neurons during OGD should be one kind of stress response to protect cells against ischemic stress (Figure 1), while the reduced expression and cytoplasmic translocation of Pnn during reoxygenation may reflect the dysfunction of Pnn and ongoing cell death. Moreover, the cytoplasmic translocation of Pnn in neurons may be caused by the increased nuclear envelop permeability, which is usually observed in apoptotic cells [25]. Of interest, the translocation of nuclear Pnn was also reported in corneal epithelial cells with hypoxia treatment [26], but not reported in other types of cells.

In addition to the interaction with mRNA slicing regulators in the nucleus, Pnn was also found to interact with a histone deacetylase complex subunit (SAP18) and regulate nonsense-mediated decay of mRNA through forming a protein complex named apoptosis-and splicing-associated protein (ASAP) [9], which was reported to participate in RNA processing and apoptosis [27]. In this study, we observe the cell type-specific intracellular distribution of SAP18. OGD treatment leads to cytoplasmic translocation of SAP18 in neurons, but not in astrocytes (Figure 1). Of interest, a more significant co-localization of Pnn and SAP18 was only observed in astrocytes with OGD, indicating that the function of ASAP may be lost in OGD-treated neurons, but enhanced in astrocytes with OGD and regeneration. Hence, the difference in Pnn/SAP18 interaction and distribution is associated with the distinct stress response in neurons and astrocytes and may determine the fate of cells post ischemic stress. This finding echoes a previous finding that indicates that ASAP and Pnn-containing ASAP (PSAP) play distinct roles in regulating mRNA alternative splicing [9]. In addition, SAP18 was also found to be a key player in transcriptional response to stress in *Drosophila* [28]. In astrocytes with OGD/R treatment, the nuclear distribution of Pnn and its co-localization with SAP18 may imply the functional maintenance of Pnn in mRNA splicing regulation on certain genes, particularly in that involved in stress response. A previous study has demonstrated that Pnn could regulate the RNA isoform expression of apoptosis-associated genes, such as Bcl-x [1]. It is reasonable that the co-localization of Pnn and SAP18 plays a protective role in astrocytes against OGD/R through mRNA alternative splicing in stress response genes.

On the contrary, the loss of co-localization of Pnn and SAP18 may render the functional alteration of ASAP and is associated with the process of apoptosis in neurons with OGD/R treatment. In addition to the role in regulating mRNA alternative splicing, Pnn was also found to be linked to the activation of ERK signaling for cellular proliferation and stress response in astrocytes and hepatic cancer cells [7,16]. However, further studies are needed to further clarify the role of Pnn and its downstream pathways in neurons with ischemic stress.

## 5. Conclusions

The Pnn expression and distribution after OGD and during reoxygenation show distinct manners in neurons and astrocytes, implying that Pnn may have different roles in different types of neural cells in the stress response to ischemic injury.
